# Perspectives in clinical research on acupuncture treatment for central obesity: A perspective

**DOI:** 10.1097/MD.0000000000042634

**Published:** 2025-05-23

**Authors:** Xi-Wen Yu, Cheng-Si Wang, Xi-Ze Sun, Jia-Mei Wu

**Affiliations:** a Department of Acupuncture and Moxibustion, Baicheng Medical College, Baicheng, China; b College of Mathematical Sciences, Shanghai Jiaotong University, Shanghai, China; c School of Chinese Medicine, Baicheng Medical College, Baicheng, China; d School of Basic Medicine, Baicheng Medical College, Baicheng, China.

**Keywords:** acupuncture, central obesity, electroacpuncture, laser acupuncture, progress

## Abstract

This study evaluates acupuncture as a viable treatment for central obesity, noting its significant effects on reducing body weight and improving metabolic health. Clinical trials attribute these benefits to acupuncture’s impact on hormonal regulation, neurological responses, and inflammation reduction. Despite its proven efficacy and minimal side effects, which promote high patient satisfaction and adherence, challenges remain. These include variability in research designs and a general lack of methodological rigor. Future research should focus on personalized treatments and integrating acupuncture with conventional methods to enhance effectiveness. Policy recommendations include incorporating acupuncture into standard obesity management protocols and training providers to ensure safe, effective treatment delivery. This holistic approach could significantly improve obesity management within contemporary healthcare settings.

## 1. Introduction

### 1.1. Overview of central obesity

Central obesity is characterized by an excessive accumulation of abdominal fat, distinct from general obesity, and is recognized by specific anthropometric measures such as waist circumference and waist-to-hip ratio.^[1]^ When these measures exceed certain thresholds, they indicate a high amount of visceral fat, which is closely linked to various metabolic syndromes including type 2 diabetes, hypertension, and cardiovascular diseases.^[2,3]^ This condition represents not only a significant health risk but also imposes substantial burdens on global healthcare systems.

### 1.2. Limitations of conventional treatments

Traditional approaches to managing central obesity focus on lifestyle interventions, including diet modifications and increased physical activity, aimed at reducing caloric intake and promoting weight loss.^[4]^ When lifestyle changes prove insufficient, pharmacological treatments may be introduced, which often involve medications designed to suppress appetite or reduce fat absorption.^[5]^ However, these methods can be limited by issues of efficacy, adherence, and sustainability, and may pose risks of adverse effects, especially with long-term use.

### 1.3. Introduction to acupuncture as an alternative approach

Acupuncture, a fundamental component of traditional Chinese medicine (TCM), is proposed as an alternative or complementary treatment for a variety of conditions, such as pain, stroke rehabilitation, cognitive impairment, Alzheimer disease diabetes mellitus, and obesity.^[6–18]^ This technique involves the strategic insertion of fine needles into designated points on the body to stimulate physiological processes and the flow of *Qi* (vital energy).^[17,18]^ In both historical and modern applications, acupuncture has been utilized to manage obesity and its related complications.

### 1.4. Potential mechanisms of acupuncture in treating central obesity

Acupuncture is believed to promote weight loss and improve fat distribution by enhancing metabolic functions, regulating hormone levels that control appetite and satiety, and reducing inflammatory responses.^[19,20]^ This modality not only targets specific symptoms or biological pathways but offers a holistic approach to restore and balance overall bodily functions.

### 1.5. Significance and scope

This introduction paves the way for a detailed exploration of the mechanisms by which acupuncture can influence metabolic and physiological parameters in central obesity. It highlights the potential of acupuncture to serve as a valuable adjunct or alternative in obesity management strategies, addressing both the limitations of conventional therapies and the complex, multifactorial nature of obesity.

## 2. Theoretical basis for acupuncture in treating central obesity

### 2.1. TCM perspective on obesity

TCM interprets obesity as a manifestation of imbalance within the body’s energetic pathways, specifically focusing on the spleen and liver organ systems.^[21]^ The spleen is vital for digesting food and transforming it into energy and essential body fluids. Conversely, the liver is responsible for regulating the flow of *Qi* and *Blood*.^[22]^ In TCM, obesity is often associated with “*Dampness*” and “*Phlegm*,” which result from a spleen deficiency that leads to poor metabolism and excessive fat storage.^[22]^

### 2.2. Mechanisms of action: acupuncture’s influence on metabolism and fat distribution

Acupuncture is believed to counteract central obesity by restoring the balance and flow of *Qi*, thereby enhancing the body’s natural metabolic processes.^[19]^ This treatment modality involves the insertion of needles at specific acupoints to stimulate the body’s healing responses. Acupuncture’s effect extends to improving the spleen’s transport and transformation functions, reducing fat accumulation through enhanced metabolic efficiency, and promoting the liver’s role in ensuring smooth *Qi* circulation.^[19]^ This can lead to a more balanced hormonal environment, increasing the metabolic rate, and regulating appetite and energy utilization effectively.

### 2.3. Review of meridians and acupoints commonly targeted for obesity

Acupuncture’s strategy in addressing obesity involves targeting meridians associated with metabolic and digestive functions:

Spleen meridian: key points such as SP6 (Sanyinjiao) and SP9 (Yinlingquan) are targeted to strengthen the spleen *Qi*, crucial for metabolic efficiency and reducing susceptibility to obesity.^[19]^

Stomach meridian: acupoints like ST36 (Zusanli) are stimulated to enhance digestive health and energy levels, facilitating weight reduction.^[23,24]^

Liver meridian: the stimulation of points such as LR3 (Taichong) helps in managing the emotional factors related to eating, associated with stress and liver *Qi* stagnation.^[23,24]^

Specific acupoints like GV26 (Shuigou) and CV12 (Zhongwan) are also emphasized for their roles in directly stimulating metabolic activity and regulating hormonal and nervous system functions.^[23,24]^ The choice of acupoints and the technique of acupuncture are tailored to each patient’s unique set of imbalances, promoting a customized approach to treatment.

Acupuncture addresses both the physiological and psycho-emotional dimensions of obesity, providing a comprehensive therapy that not only facilitates effective weight management but also enhances overall health by restoring systemic balance.^[25]^ This integrative treatment model positions acupuncture as a potent adjunct or alternative to conventional obesity management strategies, promoting sustained health benefits and improved quality of life.

## 3. Methodological approaches in acupuncture research

### 3.1. Description of common research designs in acupuncture studies

In acupuncture research, a variety of study designs are employed to assess efficacy and elucidate mechanisms.^[26–28]^ The gold standard among these is the randomized controlled trial (RCT), which reduces bias by randomly assigning participants to an intervention group receiving acupuncture or a control group.^[26]^ Often, RCTs incorporate sham acupuncture (using non-penetrating needles or placing needles at non-acupoints) to distinguish between the specific effects of acupuncture and general placebo effects derived from patient expectations or the therapeutic environment.^[27,28]^ Cohort studies also play a crucial role by following groups of patients over time to observe long-term outcomes of acupuncture compared to those who receive no treatment or alternative therapies. Although less common, case–control studies provide insights into rarer conditions or complex etiologies that are not easily addressed through other methods.

### 3.2. Discussion of methodological challenges and advances

Methodological challenges in acupuncture research mainly arise from the highly individualized nature of the treatment, the complexities in designing effective placebo controls, and the reliance on subjective outcome measures such as pain relief or improvement in quality of life.^[26]^ For instance, Vickers et al^[29]^ employed an advanced sham acupuncture technique designed to closely simulate the acupuncture procedure without stimulating the specific acupoints. This method effectively controlled for placebo effects, thereby improving the reliability of the research outcomes. By employing this approach, the study provided valuable insights into distinguishing between the physiological effects of acupuncture and the placebo response, enhancing the credibility of the findings.

The traditional RCT framework often struggles to accommodate the personalized and holistic aspects of acupuncture.^[26,29]^ Lu et al^[27]^ conducted a pragmatic trial to assess the effectiveness of acupuncture in real-world clinical settings. This approach addressed the limitations inherent in conventional RCT designs by providing a more flexible framework that accommodates the individualized nature of acupuncture treatment. The findings from this trial offer practical evidence that aligns more closely with routine clinical practices, thereby increasing the external validity of the research and making the results more applicable to day-to-day patient care. In response, the field has seen methodological advancements including the development of sophisticated sham acupuncture techniques, which aim to better mimic the procedure without activating specific acupoints, and the introduction of pragmatic trials that evaluate the treatment’s effectiveness in real-world settings, thus mirroring actual clinical practices. These pragmatic trials are invaluable for translating research findings into practice, offering insights that are readily applicable to patient care.

### 3.3. Criteria for evaluating the quality of acupuncture research

The quality of acupuncture research is assessed through several stringent criteria.^[26,29–31]^ First, the design and execution of the study must be rigorous, with a clear description of randomization, blinding methods, and participant selection criteria.^[26,29]^ Effective control measures, particularly the use of appropriate sham procedures, are essential to isolate the physiological effects of acupuncture from placebo responses.^[30,31]^Outcome measures should be clearly defined, relevant, and measurable using validated instruments. The adequacy of sample sizes is crucial to ensure that the studies have sufficient statistical power and that their findings can be generalized.^[30,31]^ Transparency in the reporting of results, including any adverse events and participant dropouts, is also critical for evaluating the safety and efficacy of the treatment. Lastly, publication in peer-reviewed journals, which requires rigorous scrutiny by field experts, serves as a benchmark for research quality.^[31]^

By enhancing these methodological aspects and adhering to rigorous evaluation criteria, acupuncture research can strengthen its evidence base, supporting its integration into contemporary therapeutic practices and enhancing its acceptance as a viable treatment option in global healthcare.

## 4. Clinical efficacy of acupuncture for central obesity

### 4.1. Summary of key clinical trials and their outcomes

The efficacy of acupuncture in treating central obesity has been demonstrated in various clinical trials. These studies typically measure outcomes such as weight loss, body mass index (BMI) reduction, changes in waist circumference (WC), and improvements in metabolic markers (Table 1).^[17,32–35]^ For instance, a RCT assessed the effects of electro-acupuncture on obese patients over an 8-week period and found that participants receiving electro-acupuncture experienced significant reductions in WC and BMI compared to those in the sham acupuncture group (Table 1).^[17]^ Similarly, a trial on laser acupuncture showed significant reductions in weight, BMI, and WC, especially in patients with periumbilical abdominal fat (Table 1).^[32]^Another study demonstrated that electro-acupuncture combined with diet control and physical exercise led to significant decreases in visceral fat thickness, BMI, and WC compared to control groups, underscoring the effectiveness of acupuncture (Table 1).^[33]^

**Table 1:** Clinical trial summary of acpuncture for patients with central obesity.

Study ref.	Patients	Treatment	Publication type	Sample size	Main findings
Lam TF, et al (2024)^[17]^	Central obesity	EA	RCT	168	EA effectively reduces waist circumference, hip circumference, body weight, BMI, and waist-to-hip ratio in central obesity
Razzaghi M, et al (2023)^[32]^	Central obesity	Laser acupuncture	RCT	64	Laser acupuncture effectively reduces periumbilical abdominal fat, offering a noninvasive weight management option
Zhong LLD, et al (2023)^[33]^	Central obesity	EA	RCT	168	EA showed superior effects in reducing waist circumference, body weight, BMI, hip circumference, waist-to-hip ratio, and body fat percentage compared to sham acupuncture
Zhang CY, et al (2015)^[34]^	Central obesity	Acupuncture	RCT	60	Acupuncture, diet, and exercise effectively reduce visceral fat and waist circumference in central obesity, with visceral fat being a key indicator of treatment success
Zhao HY. (2006)^[35]^	Central obesity	Acupuncture	Observation study	32	Acupuncture combined with electro-acupuncture, ear point tapping, and TDP radiation enhances the therapeutic effects on central obesity

BMI = body mass index, EA = electro-acupuncture, RCT = randomized controlled trial.

### 4.2. Comparative analysis of acupuncture vs other treatments

When comparing acupuncture to other obesity treatments, such as lifestyle modifications and pharmacological interventions, acupuncture often shows comparable efficacy in weight reduction and offers advantages in sustainability and side effect profiles (Table 1).^[34]^ While lifestyle changes are fundamental to obesity management, maintaining these changes can be challenging for many patients.^[34]^ Acupuncture supports these changes by reducing appetite and enhancing satiety through hormonal regulation (Table 1).^[35]^ In contrast, pharmacological treatments can lead to significant side effects, which acupuncture avoids, making it a safer long-term option.

### 4.3. Meta-analyses and systematic reviews findings

Meta-analyses and systematic reviews provide a comprehensive overview of the research, aggregating data from multiple studies to assess the overall efficacy of acupuncture for central obesity.^[24,25,36,37]^ A meta-analysis reviewed several randomized controlled trials and concluded that acupuncture significantly improves outcomes for patients with obesity, particularly in reducing WC and BMI compared to no treatment or sham acupuncture.^[36,37]^ Furthermore, combining acupuncture with lifestyle interventions was found to enhance the effectiveness of these conventional approaches, suggesting a synergistic effect.^[37]^

These findings highlight the potential of acupuncture as a viable option for managing central obesity, especially when integrated with other treatment modalities.^[34]^ Acupuncture’s ability to provide additional metabolic benefits without the adverse effects commonly associated with pharmacological treatments makes it an attractive complementary approach.^[19,25]^ However, further high-quality, large-scale studies are needed to consolidate acupuncture’s role in obesity management and to refine the protocols for its use in clinical settings. Such research will help in defining optimal treatment frequencies, durations, and specific acupoint combinations that are most effective for patients suffering from central obesity.

## 5. Mechanisms underlying acupuncture’s effects on central obesity

### 5.1. Insights from biomedical research: hormonal changes, appetite regulation, and energy expenditure

**Figure 1.**
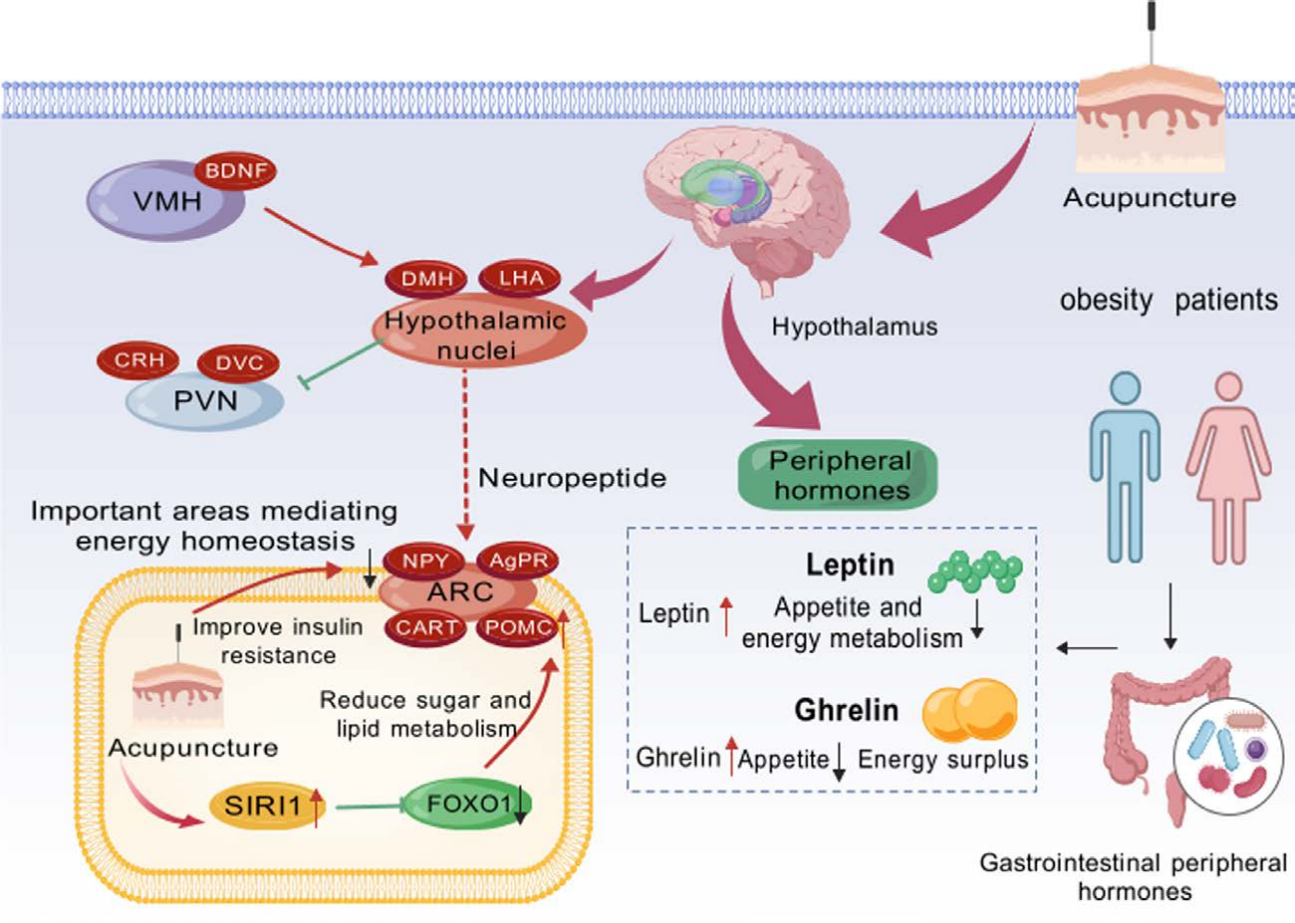
The effect of acupuncture on changes of leptin and ghrelin.

Biomedical studies have elucidated the impact of acupuncture on central obesity through its modulation of hormonal pathways. Acupuncture has been documented to influence the levels of leptin, a hormone that suppresses appetite, and ghrelin, which stimulates hunger.^[19]^ To enhance the understanding of these hormonal changes, we have expanded the discussion to include the underlying biological mechanisms through which acupuncture affects leptin and ghrelin secretion^[36,38]^ (Fig. 1). Specifically, acupuncture stimulation of certain acupoints has been shown to modulate hypothalamic activity, which in turn regulates the release of these hormones. Research demonstrates that acupuncture treatments can elevate leptin concentrations and decrease ghrelin levels, leading to a reduction in appetite and an increase in satiety.^[36,38]^ (Fig. 1). Furthermore, these treatments have been associated with improved insulin sensitivity, which is crucial for the regulation of glucose levels and fat storage.^[36,38]^ Such hormonal adjustments are instrumental in diminishing caloric intake and boosting metabolic rate, thereby facilitating the reduction of body fat, particularly in the abdominal region.

### 5.2. Neurological mechanisms: impact on the nervous system and brain centers

The neurological effects of acupuncture on obesity management are significant and complex.^[19,39,40]^ Neuroimaging research has shown that acupuncture activates specific areas of the brain involved in hunger and satiety regulation, including the hypothalamus and limbic system.^[19,39,40]^ The procedure stimulates the release of endorphins and other neurotransmitters that influence mood and appetite. These effects help to regulate eating behaviors and are crucial for supporting the lifestyle changes necessary for effective weight management.

### 5.3. Role of inflammation and oxidative stress in obesity: acupuncture’s modulatory effects

Chronic inflammation and oxidative stress play critical roles in the pathology of obesity and its complications, such as metabolic syndrome. Acupuncture has demonstrated potential anti-inflammatory and antioxidative effects.^[41,42]^ Clinical studies have noted reductions in inflammatory markers like C-reactive protein and tumor necrosis factor-alpha following acupuncture treatment.^[42]^ Similarly, enhancements in the body’s antioxidant capacity have been observed, contributing to decreased oxidative stress. These effects not only aid in mitigating the metabolic imbalances associated with obesity but also promote broader health improvements.

The mechanisms by which acupuncture affects central obesity are thus characterized by a dynamic interplay between hormonal regulation, neural activity, and anti-inflammatory actions.^[42]^ These scientifically supported interactions provide a strong rationale for the use of acupuncture as an adjunctive or alternative therapy in obesity management. Further exploration and refined research methodologies are needed to optimize acupuncture protocols and fully harness its therapeutic potential for treating central obesity.

## 6. Safety, side effects, and patient acceptability of acupuncture for obesity

### 6.1. Review of safety data from clinical trials

Acupuncture is recognized as a safe modality when administered by qualified professionals.^[43,44]^ Detailed analyses of clinical trials emphasize that serious complications are exceedingly rare.^[43,44]^ To further substantiate this safety profile, we have incorporated additional studies along with a recent meta-analysis focusing on acupuncture safety outcomes.^[43,44]^ By aggregating data from multiple clinical trials, these meta-analyses provide a comprehensive understanding of the associated risks, reinforcing acupuncture’s safety when administered by trained practitioners.^[45,46]^ This evidence not only strengthens the discussion on minimal adverse effects but also supports the high patient acceptability of acupuncture in obesity management.

The most thorough safety evaluations suggest that the typical side effects are minor, including symptoms like bruising, slight bleeding at the insertion points, and occasional mild pain or discomfort during the procedure.^[43,44]^ The risk of infections is notably low, contingent on practitioners adhering to stringent sterilization protocols and utilizing disposable needles.

### 6.2. Common side effects and their management

Although acupuncture is safe for the majority of patients, some common side effects are noted.^[43,44]^ These generally include temporary bruising and discomfort at needle sites, dizziness, and infrequently, fainting. Such effects are usually mild and short-lived.^[43,44]^ Managing these symptoms effectively involves ensuring patient comfort during the session, selecting appropriate needle sizes, and providing clear aftercare instructions. Acupuncturists are trained to closely observe patients for any signs of discomfort during the treatment and to modify the treatment approach if necessary.

### 6.3. Patient perspectives on acupuncture treatment for obesity

Patient acceptance and satisfaction with acupuncture for obesity management are typically positive.^[47,48]^ Many patients prefer acupuncture over pharmacological alternatives due to its minimal side effects and noninvasive nature.^[47]^ Qualitative research indicates that patients value the enhanced sense of well-being and relaxation afforded by acupuncture sessions.^[48]^ Additionally, many report ancillary benefits such as improved stress management and increased energy levels, attributing these improvements to the holistic approach of acupuncture.

Adherence to acupuncture treatment is generally strong, attributed to the personalized nature of the therapy and the strong therapeutic rapport between patient and practitioner. Patients’ commitment to the treatment and broader lifestyle adjustments is often reinforced by the educational support provided by acupuncturists, who explain the treatment process and expected outcomes in detail.^[47,48]^

In summary, acupuncture emerges as a safe, effective, and highly acceptable treatment for obesity, characterized by minimal and manageable side effects, alongside high levels of patient satisfaction and adherence. These aspects make acupuncture a compelling alternative or supplementary treatment to conventional methods. Ongoing research focused on long-term safety, efficacy, and patient-centered outcomes will further solidify acupuncture’s role in integrated obesity management strategies.

## 7. Challenges and future directions in acupuncture research for obesity

Despite a growing body of evidence supporting acupuncture’s efficacy in treating obesity, significant research gaps persist, particularly concerning the consistency of study designs. Many studies exhibit variability that complicates the comparison of results and undermines the overall cohesion of the evidence base.^[36,37]^ Moreover, there is often a lack of rigorous methodological standards, such as sufficient sample sizes and well-constructed control groups.^[36]^ Additionally, the underlying mechanisms through which acupuncture affects metabolic processes and fat distribution are not fully understood and are frequently based more on theoretical speculation than on solid empirical evidence.^[19]^

The potential for integrating acupuncture with other obesity treatments presents a promising avenue for enhancing treatment efficacy.^[36]^ By combining acupuncture with conventional treatment modalities, such as dietary modifications, physical activity, and behavioral therapy, a more comprehensive, holistic approach to obesity management can be achieved.^[34,35]^ This approach would not only adhere to the holistic principles of TCM, which focus on treating the individual as a whole, but could also lead to synergistic effects, potentially resulting in more significant and sustained weight loss.^[49]^ Research into the efficacy of these integrated treatment protocols is essential for developing optimized, multimodal therapeutic strategies.

To advance the field of acupuncture for obesity, several critical research areas must be addressed. By focusing on these priorities, researchers can contribute to the development of standardized and effective acupuncture protocols that enhance treatment outcomes across diverse patient populations and facilitate its integration into contemporary obesity management strategies.

Optimization of treatment protocols: future research should focus on determining the optimal parameters for acupuncture treatment, such as the frequency, duration, and specific acupoint combinations that are most effective for weight management. Investigating how these protocols can be tailored to address the unique needs of various patient demographics, including differences in age, sex, and metabolic conditions, will further enhance their applicability. By establishing evidence-based guidelines, researchers can ensure more consistent and effective clinical applications of acupuncture.^[36,37,47]^

Personalization of treatments: exploring how acupuncture can be customized to individual patient characteristics represents a crucial area for future investigation. Genetic predispositions, metabolic profiles, and behavioral patterns may all influence a patient’s response to acupuncture. Personalized approaches that consider these factors could align with broader trends in precision medicine, enabling treatments to be more targeted and effective, ultimately improving patient outcomes.^[24]^

Long-term effects and sustainability: the durability of acupuncture’s effects on weight management requires comprehensive evaluation through longitudinal studies. Future research should aim to identify the maintenance treatment frequency and duration necessary to sustain long-term benefits, such as continued weight loss and improved metabolic health. Furthermore, these studies should assess the potential synergistic effects of integrating acupuncture with lifestyle interventions, such as dietary modifications and physical activity, to ensure sustainable outcomes.^[50]^

Mechanistic studies: a deeper understanding of the mechanisms through which acupuncture influences obesity is essential to refine its therapeutic application. Research should explore how acupuncture affects the nervous system, modulates hormonal pathways, including the regulation of leptin and ghrelin, and mitigates inflammation and oxidative stress. Mechanistic insights from these studies will not only clarify the biological basis of acupuncture’s effects but also guide the development of complementary therapeutic strategies.^[19,41]^

Standardization and integration: the standardization of acupuncture protocols is critical to its broader acceptance in clinical practice. Future studies should aim to establish reproducible treatment methodologies and validate these protocols across diverse clinical settings. Standardized practices will facilitate the integration of acupuncture into conventional obesity management strategies, ensuring it is recognized as a reliable and effective therapeutic option. This integration will also enhance the credibility of acupuncture within the global healthcare community and promote its acceptance among practitioners and policymakers.^[36,37]^

## 8. Summary

This analysis underscores the role of acupuncture as a viable treatment for central obesity, demonstrating through clinical trials that it can significantly decrease body weight and improve metabolic parameters. The mechanisms underlying these effects, hormonal adjustments, neurological modulation, and reduction in inflammation, illustrate acupuncture’s capacity to address complex aspects of obesity comprehensively.

These findings suggest important implications for clinical practice. Incorporating acupuncture into broader obesity management strategies can potentially enhance treatment outcomes due to its efficacy and safety profile. For healthcare policy, these results support the recommendation to include acupuncture within health insurance coverage, making it more accessible as a standard therapeutic option for obesity. This policy shift could encourage a more integrative approach to obesity treatment, emphasizing patient-centered care.

Additionally, ensuring that healthcare providers receive proper training in acupuncture techniques will be crucial for safe and effective treatment delivery. Adopting these measures will not only facilitate the integration of acupuncture into conventional medical settings but also promote a diversified approach to obesity management that aligns with modern healthcare principles.

## Author contributions

**Conceptualization:** Xi-Wen Yu, Cheng-Si Wang, Jia-Mei Wu.

**Data curation:** Xi-Wen Yu, Cheng-Si Wang, Xi-Ze Sun, Jia-Mei Wu.

**Investigation:** Jia-Mei Wu.

**Methodology:** Xi-Wen Yu, Cheng-Si Wang.

**Project administration:** Jia-Mei Wu.

**Resources:** Xi-Wen Yu, Xi-Ze Sun.

**Supervision:** Jia-Mei Wu.

**Validation:** Xi-Wen Yu, Cheng-Si Wang, Xi-Ze Sun, Jia-Mei Wu.

**Visualization:** Xi-Wen Yu, Cheng-Si Wang, Xi-Ze Sun, Jia-Mei Wu.

**Writing – original draft:** Xi-Wen Yu, Cheng-Si Wang, Xi-Ze Sun, Jia-Mei Wu.

**Writing – review & editing:** Xi-Wen Yu, Cheng-Si Wang, Xi-Ze Sun, Jia-Mei Wu.

## References

[1] Klein S Allison DB Heymsfield SB. Waist circumference and cardiometabolic risk: a consensus statement from Shaping America’s Health: association for Weight Management and Obesity Prevention; NAASO, The Obesity Society; the American Society for Nutrition; and the American Diabetes Association. Am J Clin Nutr. 2007;85:1197–202. 17490953 10.1093/ajcn/85.5.1197

[2] Siren R Eriksson JG Vanhanen H. Waist circumference a good indicator of future risk for type 2 diabetes and cardiovascular disease. BMC Public Health. 2012;12:631. 22877354 10.1186/1471-2458-12-631 PMC3490795

[3] Al-Hamodi Z Ismail IS Saif-Ali R Ahmed KA Muniandy S. Association of plasminogen activator inhibitor-1 and tissue plasminogen activator with type 2 diabetes and metabolic syndrome in Malaysian subjects. Cardiovasc Diabetol. 2011;10:23. 21414238 10.1186/1475-2840-10-23 PMC3064636

[4] Madigan CD Graham HE Sturgiss E. Effectiveness of weight management interventions for adults delivered in primary care: systematic review and meta-analysis of randomised controlled trials. BMJ. 2022;377:e069719. 35636762 10.1136/bmj-2021-069719 PMC9150078

[5] Kosmalski M Deska K Bąk B Różycka-Kosmalska M Pietras T. Pharmacological support for the treatment of obesity-present and future. Healthcare (Basel). 2023;11:433. 36767008 10.3390/healthcare11030433 PMC9914730

[6] Niruthisard S Ma Q Napadow V. Recent advances in acupuncture for pain relief. Pain Rep. 2024;9:e1188. 39285954 10.1097/PR9.0000000000001188 PMC11404884

[7] Zhang QH Yue JH Sun ZR Lu Y. Acupuncture for chronic knee pain: a protocol for an updated systematic review. BMJ Open. 2016;6:e008027. 10.1136/bmjopen-2015-008027 PMC4769398 26911581

[8] Zhang Q Yue J Zeng X Sun Z Golianu B. Acupuncture for chronic neck pain: a protocol for an updated systematic review. Syst Rev. 2016;5:76. 27146261 10.1186/s13643-016-0257-x PMC4857250

[9] Yue JH Li A Cui X. Bibliometric analysis of acupuncture for headache from 1974 to 2022: A scoping literature review based on international database. Medicine (Baltimore). 2023;102:e34590. 37543789 10.1097/MD.0000000000034590 PMC10402990

[10] Lee JE Akimoto T Chang J Lee HS. Effects of joint mobilization combined with acupuncture on pain, physical function, and depression in stroke patients with chronic neuropathic pain: a randomized controlled trial. PLoS One. 2023;18:e0281968. 37616239 10.1371/journal.pone.0281968 PMC10449141

[11] Yue JH Golianu B Zeng XX. Acupuncture for urinary retention after stroke: a protocol for systematic review. Eur J Bio Med Res. 2015;1:7–11.

[12] Zhang Q Liu C Jing X. Editorial: Neural mechanism and effect of acupuncture for central nervous system diseases. Front Neurosci. 2024;17:1337612. 38260027 10.3389/fnins.2023.1337612 PMC10800381

[13] Lin G Yie SLJ Guo S Li X Xu L. Clinical evidence of acupuncture for amnestic mild cognitive impairment: a systematic review and meta-analysis of randomized controlled trials. Complement Ther Med. 2024;103114. 10.1016/j.ctim.2024.103114 39617303

[14] Yue J Li XL Gao RX. Research status, hotspots and trends of acupuncture and moxibustion in the treatment of Alzheimer’s disease: a bibliometric analysis. Medicine (Baltimore). 2022;101:e30858. 36181105 10.1097/MD.0000000000030858 PMC9524865

[15] Hoerder S Habermann IV Hahn K. Acupuncture in diabetic peripheral neuropathy-neurological outcomes of the randomized acupuncture in diabetic peripheral neuropathy trial. World J Diabetes. 2023;14:1813–23. 38222786 10.4239/wjd.v14.i12.1813 PMC10784801

[16] He K Zhang Q Yue J. Research progress in molecular mechanism of acupuncture for diabetes mellitus. Zhongguo Zhen Jiu. 2024;44:1357–62. 39532456 10.13703/j.0255-2930.20240604-0003

[17] Lam TF Lyu Z Wu X. Electro-acupuncture for central obesity: a patient-assessor blinded, randomized sham-controlled clinical trial. BMC Complement Med Ther. 2024;24:62. 38287303 10.1186/s12906-024-04340-5 PMC10823622

[18] Belivani M Dimitroula C Katsiki N Apostolopoulou M Cummings M Hatzitolios AI. Acupuncture in the treatment of obesity: a narrative review of the literature. Acupunct Med. 2013;31:88–97. 23153472 10.1136/acupmed-2012-010247

[19] Wang L Yu CC Li J Tian Q Du YJ. Mechanism of action of acupuncture in obesity: a perspective from the hypothalamus. Front Endocrinol (Lausanne). 2021;12:632324. 33868169 10.3389/fendo.2021.632324 PMC8050351

[20] Jiang LY Tian J Yang YN Jia SH Shu Q. Acupuncture for obesity and related diseases: insight for regulating energy metabolism and neural circuits. J Integr Med. 2024;22:93–101. 38519278 10.1016/j.joim.2024.03.001

[21] Ding X He X Tang BL Lan T. Integrated traditional Chinese and Western medicine in the prevention and treatment of non-alcoholic fatty liver disease: future directions and strategies. Chin Med. 2024;19:21. 38310315 10.1186/s13020-024-00894-1 PMC10838467

[22] Zhang CH Sheng JQ Xie WH. Mechanism and basis of traditional Chinese medicine against obesity: Prevention and treatment strategies. Front Pharmacol. 2021;12:615895. 33762940 10.3389/fphar.2021.615895 PMC7982543

[23] Lu PH Chen YY Tsai FM. Combined acupoints for the treatment of patients with obesity: an association rule analysis. Evid Based Complement Alternat Med. 2022;2022:7252213. 35341146 10.1155/2022/7252213 PMC8947926

[24] Chen J Shergis JL Guo X. Acupuncture therapies for individuals with overweight or obesity: an overview of systematic reviews. Diabetes Metab Syndr Obes. 2022;15:1651–66. 35669360 10.2147/DMSO.S356853 PMC9165609

[25] Zhang Y Li J Mo G. Acupuncture and related therapies for obesity: a network meta-analysis. Evid Based Complement Alternat Med. 2018;2018:9569685. 30363899 10.1155/2018/9569685 PMC6186334

[26] MacPherson H Altman DG Hammerschlag R. Revised STandards for Reporting Interventions in Clinical Trials of Acupuncture (STRICTA): extending the CONSORT statement. J Evid Based Med. 2010;3:140–55. 21349059 10.1111/j.1756-5391.2010.01086.x

[27] Lu L Zhang Y Tang X. Evidence on acupuncture therapies is underused in clinical practice and health policy. BMJ. 2022;376:e067475. 35217525 10.1136/bmj-2021-067475 PMC8868048

[28] Zhang YQ Jiao RM Witt CM. How to design high quality acupuncture trials – a consensus informed by an international panel. BMJ. 2022;376:e067476. 35354583 10.1136/bmj-2021-067476 PMC8965655

[29] Vickers AJ Vertosick EA Lewith G. Acupuncture for chronic pain: update of an individual patient data meta-analysis. J Pain. 2018;19:455–74. 29198932 10.1016/j.jpain.2017.11.005 PMC5927830

[30] Langevin HM Wayne PM. What is the point? The problem with acupuncture research that no one wants to talk about. J Altern Complement Med. 2018;24:200–7. 29493256 10.1089/acm.2017.0366 PMC6421999

[31] Manheimer E Wieland S Kimbrough E Cheng K Berman BM. Evidence from the Cochrane collaboration for traditional Chinese medicine therapies. J Altern Complement Med. 2009;15:1001–14. 19757977 10.1089/acm.2008.0414 PMC2856612

[32] Razzaghi M Akbari Z Mokmeli S. Laser diode – GaAlAs acupuncture in the treatment of central obesity: a randomized clinical trial. J Acupunct Meridian Stud. 2023;16:255–62. 38115591 10.51507/j.jams.2023.16.6.255

[33] Zhong LLD Zhang S Wong E Cao C Bian ZX. Electro-acupuncture for central obesity: abridged secondary publication. Hong Kong Med J. 2023;29(Suppl 2):33–4. 36951004

[34] Zhang CY Yang L. Effect of acupuncture therapy on visceral fat thickness in simple central obesity patients. Zhen Ci Yan Jiu. 2015;40:484–8. 26887212

[35] Zhao HY. Clinical observation on acupuncture for treatment of central obesity. Zhongguo Zhen Jiu. 2006;26:629–31. 17036479

[36] Cho SH Lee JS Thabane L Lee J. Acupuncture for obesity: a systematic review and meta-analysis. Int J Obes (Lond). 2009;33:183–96. 19139756 10.1038/ijo.2008.269

[37] Zhang RQ Tan J Li FY Ma YH Han LX Yang XL. Acupuncture for the treatment of obesity in adults: a systematic review and meta-analysis. Postgrad Med J. 2017;93:743–51. 28689171 10.1136/postgradmedj-2017-134969

[38] Wang LL Yin GZ. Effects of acupuncture on leptin level and relative factors in the simple obesity Uigur patient. Zhongguo Zhen Jiu. 2005;25:834–6. 16419701

[39] Dhond RP Kettner N Napadow V. Neuroimaging acupuncture effects in the human brain. J Altern Complement Med. 2007;13:603–16. 17718643 10.1089/acm.2007.7040

[40] Hui KK Liu J Makris N. Acupuncture modulates the limbic system and subcortical gray structures of the human brain: evidence from fMRI studies in normal subjects. Hum Brain Mapp. 2000;9:13–25. 10643726 10.1002/(SICI)1097-0193(2000)9:1<13::AID-HBM2>3.0.CO;2-F PMC6871878

[41] Wang LH Huang W Wei D. Mechanisms of acupuncture therapy for simple obesity: an evidence-based review of clinical and animal studies on simple obesity. Evid Based Complement Alternat Med. 2019;2019:5796381. 30854010 10.1155/2019/5796381 PMC6378065

[42] Abdi H Zhao B Darbandi M. The effects of body acupuncture on obesity: anthropometric parameters, lipid profile, and inflammatory and immunologic markers. ScientificWorldJ. 2012;2012:603539. 10.1100/2012/603539 PMC3353309 22649299

[43] Witt CM Pach D Brinkhaus B. Safety of acupuncture: results of a prospective observational study with 229,230 patients and introduction of a medical safety network. Forsch Komplementmed. 2009;16:91–7. 19420954 10.1159/000209315

[44] White A Hayhoe S Hart A Ernst E. Adverse events following acupuncture: prospective survey of 32 000 consultations with doctors and physiotherapists. BMJ. 2001;323:485–6. 11532840 10.1136/bmj.323.7311.485 PMC48133

[45] Xu S Wang L Cooper E. Adverse events of acupuncture: a systematic review of case reports. Evid Based Complement Alternat Med. 2013;2013:581203. 23573135 10.1155/2013/581203 PMC3616356

[46] Lao L Hamilton GR Fu J Berman BM. Is acupuncture safe? A systematic review of case reports. Altern Ther Health Med. 2003;9:72–83. 12564354

[47] Jakes D Kirk R Muir L. A qualitative systematic review of patients’ experiences of acupuncture. J Altern Complement Med. 2014;20:663–71. 25072404 10.1089/acm.2013.0446

[48] Chiolero A Burnier M Santschi V. Improving treatment satisfaction to increase adherence. J Hum Hypertens. 2016;30:295–6. 26290276 10.1038/jhh.2015.89

[49] Sui Y Zhao HL Wong VC. A systematic review on use of Chinese medicine and acupuncture for treatment of obesity. Obes Rev. 2012;13:409–30. 22292480 10.1111/j.1467-789X.2011.00979.x

[50] Dai L Wang M Zhang KP. Modified acupuncture therapy, long-term acupoint stimulation versus sham control for weight control: a multicenter, randomized controlled trial. Front Endocrinol (Lausanne). 2022;13:952373. 35966092 10.3389/fendo.2022.952373 PMC9365970

